# Anti-Oxidative Activity of Alcohol-Water Extracts from Field Horsetail (*Equisteum arvense*) in Elastomer Vulcanizates Subjected to Accelerated Aging Processes

**DOI:** 10.3390/ma13214903

**Published:** 2020-10-31

**Authors:** Marcin Masłowski, Justyna Miedzianowska, Agnieszka Czylkowska, Magdalena Efenberger-Szmechtyk, Agnieszka Nowak, Krzysztof Strzelec

**Affiliations:** 1 Institute of Polymer & Dye Technology, Lodz University of Technology, Stefanowskiego 12/16, 90-924 Lodz, Poland; justyna.miedzianowska@dokt.p.lodz.pl (J.M.); krzysztof.strzelec@p.lodz.pl (K.S.); 2 Institute of General and Ecological Chemistry, Lodz University of Technology, Zeromskiego 116, 90-924 Lodz, Poland; agnieszka.czylkowska@p.lodz.pl; 3 Institute of Fermentation Technology and Microbiology, Lodz University of Technology, Wolczanska 171/173, 90-924 Lodz, Poland; magdalena.efenberger-szmechtyk@edu.p.lodz.pl (M.E.-S.); agnieszka.nowak@p.lodz.pl (A.N.)

**Keywords:** horsetail, natural rubber, natural extracts, antioxidants, aging

## Abstract

The article aimed to highlight the antioxidant potential of natural extracts obtained from *Equisteum arvense* in elastomer vulcanizatec made of natural rubber. Horsetail extracts were prepared using four solvent systems at different volume ratios: methanol–water (50/50 and 70/30) and ethanol–water (50/50 and 70/30), which were then lyophilized and added to the polymer matrix. A deep characterization of the obtained bio-additives was performed. The obtained vulcanizates were subjected to various aging processes: thermo-oxidative, ultraviolet and solar. Then, the resistance and susceptibility of vulcanizates to selected processes of accelerated, simulated degradation were determined based on the changes in the spatial structure (network nodes), material strength and physical properties (color). The research showed the presence of polyphenols in the extracts of horsetail, which resulted in their high anti-oxidative activity. Moreover, the extracts were able to absorb UV radiation. As a consequence, they were active additives that protected rubber vulcanizates against the damaging effects of the aging processes, securing their functional properties. Samples containing natural extracts were characterized by better stability of: mechanical strength parameters, the increase in cross-linking density and color changes after simulating aging processes in comparison with the control sample.

## 1. Introduction

Secondary metabolites present in huge amounts in higher plants are extremely valuable components of pharmaceuticals, agrochemicals, flavors, aromas, dyes, biopesticides and food additives [1]. It has been scientifically confirmed that a wide range of biological effects, including antioxidant, antibacterial and anti-inflammatory effects, are attributable to natural phytochemicals derived from fruits, vegetables and herbs [2,3,4]. Research on phenolic acids and flavonoids proves their increased antioxidant activity. A characteristic feature of these compounds is mainly their ability to act as free radical scavengers and as metal chelators [5,6]. These kinds of compounds very likely bond with metal ions to form coordination compounds. The complexes are very stable and contribute to greater antioxidant activity of flavonoids. Some of them in the presence of copper ions may also undergo the autooxidation process [7,8]. Besides, vegetables and fruits have also been shown to contain other antioxidant nutrients in addition to the well-known vitamins C and E and carotenoids that contribute significantly to their overall antioxidant capacity [9,10,11]. Therefore, research to find natural plant-derived antioxidants is highly desirable. That is also related to the fact that they are used as an alternative to synthetic antioxidants [12].

For centuries, herbs have been used for many different purposes, including drugs, nutrition, flavorings, drinks, dyeing, repellants, fragrances, cosmetics, amulets, smoking and industrial applications. Nowadays, they are the base materials for obtaining valuable and attractive herbal extracts with various applications [13,14,15]. The field horsetail (*Equisetum arvense L*.) is a perennial of the horsetail family (Equisetaceae) common in Europe, Asia and North America. Its herb represents an exceptionally rich source of substances with a pro-health effect. Of course, it should be noted that most of this effect is carried out in a genomic manner; that of course it does not work in the present case. However, current pharmaceutical research shows that E. arvense has antioxidant, anti-inflammatory, antidiabetic, antibacterial, antifungal, anticonvulsant and anticancer effects [16,17,18]. It has been used in the treatment of various diseases, such as tuberculosis, runny nose, nail diseases and urinary tract diseases; as a hematostatic agent in nose, lung and stomach hemorrhages; and in the treatment of hair loss, rheumatic diseases, gout, poorly healing wounds, ulcers, swelling, fractures and frostbite [19]. Field horsetail owes its healing effect to its chemical structure. Apart from the over 10% of inorganic substances (most of them are silicic acid and potassium salts), it contains mainly [20]: sterols—β-sitosterol, campesterol and isofucosterol [21]; ascorbic acid [11]; phenolic acids (cinnamic, caffeic, di-E-caffeoyl-meso-tartaric and 5-O-caffeoylshikimic acids) [22]; polyunsaturated acids, rare dicarboxylic acids (equisetolic acid) and styrylpyrones [23]; and flavonoids (apigenin, kaempferol, luteolin, quercetin, glycosides and their malonyl esters; other polyphenolic compounds such as caffeic acid derivatives) [24,25].

The analysis of experimental studies shows that water and ethanol extracts of *Equisteum arvense* have very good antioxidant properties [11]. They were characterized by a strong protective effect against free radicals, lipid peroxidation and oxidizing agents. It should also be emphasized that the authors of the studies pointed out that the antioxidant activity of horsetail extracts was higher or similar to the commercial antioxidants available on the market [20]. Unfortunately, the number of scientific reports on the antioxidant properties of *Equisteum arvense* is still extremely low. Moreover, apart from the authors’ article [26], there are no studies devoted to the activity of the field horsetail in elastomer technology. The use of this herb as an active additive in natural rubber composites can be an extremely valuable contribution to the development of knowledge about its anti-aging activity.

Natural rubber (NR) is renewable, non-toxic polymer. Due to its low price, it is the most used elastomer in industry worldwide. Due to its above-average functional properties, natural rubber presents a wide range of possible applications in various sectors of the economy, for example, in the automotive, footwear, furniture, construction and construction industries [27]. Natural rubber, despite many advantages and possible applications, also has disadvantages. One of the main features hindering the long-term operation of finished products is their susceptibility to accelerated aging processes. NR has double bonds in its structure that hinder stabilization, exposing the material to uncontrolled and undesirable aging processes. In order to limit the negative effects of aging, it is necessary to use various types of stabilizers [28].

*Equisteum arvense* extracts offer the possibility of using them as natural anti-aging agents, replacing commercially available, synthetic additives. In-depth research on this topic is necessary in order to develop new, environmentally friendly technologies for elastomeric materials.

## 2. Materials and Methods

### 2.1. Materials

#### 2.1.1. Elastomer

Natural rubber (NR)–RSSI, cis-1,4-polyisoprene, density 0.93–0.98 g/cm^3^, was provided by Torimex Chemicals (Lodz, Poland).

#### 2.1.2. Cross-Linking System

The crosslinking system consisted of: sulfur (Siarkopol, Tarnobrzeg, Poland), microsized zinc oxide (Sigma-Aldrich, St. Louis, MO, USA), steric acid (Avantor Performance Materials, Gliwice, Poland) and 2-mercaptobenzothiazole (Sigma-Aldrich, St. Louis, MO, USA).

#### 2.1.3. Natural Extracts

Extraction from horsetail (*Equisetum arvense L*), provided by ManuTea (Chałupki, Poland), was performed using a Series 148 extractor from Velp (Usmate Velate, Italy); 15 g of horsetail (ground and dried) was placed in a cellulose thimble and immersed in a solvent (100 mL). Four types of ethanol–water extraction systems in 50/50 and 70/30 ratios (W/E_50/50, W/E_30/70) and methanol–water in the same ratios (W/M_50/50, W/M_30/70) were performed. The individual system was brought to a boil and the extraction process was carried out for 2 h. After that time, the thimbles were lifted over the solvent and rinsed with the evaporating solvent (step 1). Then, new thimbles containing another 15 g of horsetail were placed in the extractor. The solvent was poured into the beakers with the resulting extract so that its volume was again 100 mL. The thimbles were immersed again and the process was carried out for another 2 h from the point of reflux. The next steps were the same as in step 1 (step 2). The thimble was changed once more (step 3). After the three-stage extraction process, the solvent was evaporated by using Heidolph, Laborata 4001 (Teltow, Germany) efficient rotary evaporator.

The prepared extract was subjected to the freeze–drying process (Labconco Freezzone 2.5 Plus, Kansas City, MN, USA) in order to obtain a solid form that could be incorporated into the elastomer blend.

#### 2.1.4. Elastomer Vulcanizates

The compositions of the tested elastomer mixtures are presented in Table 1.

### 2.2. Methods

#### 2.2.1. Thermogravimetric Analysis (TGA)

The thermal stability of freeze-dried extracts (W/E_50/50, W/E_30/70, W/M_50/50, W/M_30/70) was studied using a TGA/DSC1 (Mettler Toledo, Columbus, OH, USA) analyzer. The sample weight was kept at approximately 10 mg. Extracts were heated from 25 to 600 °C with a heating rate of 10 °C/min in an argon atmosphere.

#### 2.2.2. UV–Vis Diffuse Reflectance Spectroscopy

Horsetail freeze-dried extracts were exanimated using an Evolution 201/220 UV–Visible Spectrophotometer (Thermo Scientific, Waltham, MA, USA). As a result of the research, the spectrum of absorption of radiation by the natural extracts in the range of 1100–200 nm was recorded.

#### 2.2.3. Fourier Transform Infrared Spectroscopy (FTIR)

The chemical structures of freeze-dried extracts (W/E_50/50, W/E_30/70, W/M_50/50, W/M_30/70) used in this study were characterized by Fourier transform infrared spectroscopy (Nicolet 6700 spectrophotometer, Thermo Electron) (Waltham, MA, USA). The spectrophotometer was equipped with a diamond Smart Orbit ATR sampling accessory. The spectrum was recorded in the 4000–400 cm^−1^ range (128 scans, absorption mode).

#### 2.2.4. Total Phenolic Content

The total phenolic content (TPC) of *E. arvense* extracts was analyzed using the Folin–Ciocalteu colorimetric method [29]. The reaction mixture consisted of: 100 µL of the extract; 200 µL of Folin–Ciocalteu reagent; 1 mL of 20% Na_2_CO_3_ solution; and 2 mL of distilled water. The blank sample containing 100 µL of distilled water instead of the extract was also prepared. The samples were mixed and kept at room temperature in a dark place for 1 h. After that time, the absorbance was measured at the wavelength of 765 nm against the prepared blank sample using a Cecil CE2041 spectrophotometer (Cecil Instruments Limited, Cambridge, UK). The TPC in the extracts was quantified according to a calibration curve and expressed as gallic acid equivalents (µgGAE/mL).

#### 2.2.5. Antioxidant Activity

Antioxidant activity of the extracts was measured with two free radical scavenging methods: DPPH and ABTS.

A DPPH free radical scavenging assay was performed according to the modified methods described by Meda et al. [30] and Al et al. [31]: 2.4 mg of 2,2-diphenyl-1-picrylhydrazyl (DPPH) (Sigma-Aldrich, St Louis, MO, USA) was dissolved with 100 mL of 80% ethanol. The reaction mixture contained 1.95 mL of the DPPH free radical solution and 50 µL of *E. arvense* extract. The samples were kept for 15 min in a dark place at room temperature. The absorbance was measured at the wavelength of 515 nm against 80% ethanol, using a Cecil CE2041 spectrophotometer (Cecil Instruments Limited, Cambridge, UK).

The ABTS radical scavenging assay was performed as described by Re et al. [32]. ABTS (2,20 -azinobis(3-ethylbenzothiazoline-6-sulphonic acid) (Sigma-Aldrich, St Louis, MO, USA) was dissolved in water to obtain the concentration equal to 7 mmol. K_2_S_2_O_8_ (final concentration of 2.45 mmol) was added to ABTS stock solution to obtain ABTS radical cation (ABTS+). The mixture was kept for 12–16 h in a dark place at room temperature before use. The ABTS+ solution was diluted to reach an absorbance of 0.700 measured at 734 nm. The reaction mixtures consisted of 3 mL of diluted ABTS+ solution and 30 µL of *E. arvense* extract. The absorbance was measured after 10 min at 734 nm against distilled water using a Cecil CE2041 spectrophotometer (Cecil Instruments Limited).

The antioxidant activity determined by DPPH and ABTS was quantified according to the calibration curve prepared for Trolox (6-hydroxy-2,5,7,8-tetramethylchroman-2-carboxylic acid) (Sigma-Aldrich, St Louis, MO, USA) and expressed as Trolox equivalents (µgTE/mL).

Mean values and standard deviations (SD) were calculated. One-way ANOVA with Tukey’s honestly significant differences (HSD) test (*p* < 0.05) was performed using R 3.4.0 (R Core Team, Vienna, Austria).

#### 2.2.6. Rubber Mixtures’ Preparation

Natural rubber mixtures containing horsetail extracts were prepared using a Brabender measuring mixer N50 (Duisburg, Germany) at 50–60 °C. At the beginning, natural rubber was introduced into the working chamber, which was plasticized for 4 min, after which the freeze-dried extract was added. Total mixing time was 10 min. Then, using a rolling mill, the cross-linking system was mixed into the previously prepared mixtures (rubber + extract) and rubber sheets were obtained.

#### 2.2.7. Vulcanization

The elastomeric compounds were cured in metal molds using a hydraulic press with heated shelves. The samples were pressed at 160 °C, at 15 MPa pressure, for a time of 10 min.

#### 2.2.8. Atomic Absorption Spectrometry (AAS)

For the analysis of metal (II) concentrations, both freeze-dried extracts and filled natural rubber were prepared. The contents of Co(II), Ni(II), Cd(II) and Pb(II) in solid states were determined by the F-AAS spectrometer with a continuum source of light and using air/acetylene flame (Analityk Jena, contraAA 300). Absorbances were measured at analytical spectral lines: 240.7 nm for Co(II), 232.0 nm for Ni(II), 228.8 nm for Cd(II) and 217 nm for Pb(II). Solid samples were decomposed using the Anton Paar Multiwave 3000 closed system instrument. Mineralization was carried out for 45 min at 240 ℃ under 60 bar pressure. Standard solutions of Merck (1000 mg/L) were used for the preparation of calibration curves.

#### 2.2.9. Aging Processes (UV, Thermo-Oxidative and Solar)

The vulcanizate samples were aged under three different conditions.

UV ageing was performed using a UV 2000 apparatus from Atlas (Mount Prospect, IL, USA). The UV ageing process was carried out in repeated (continuously) segments: daily segment (UV radiation intensity = 0.7 W/m^2^, temperature = 60 °C, duration = 8 h) and night segment (without UV radiation, temperature = 50 °C, duration = 4 h).

The solar aging process (SOLAR) was performed using a Solar SC 340 (Atlas, Mount Prospect, IL, USA). Parameters of aging: light radiation of λ = 280–3000 nm, temperature = 40°C, humidity = 55%.

The thermo-oxidative degradation (TO) of the natural rubber vulcanizates was carried out based on the PN-82/C-04216 standard. The TO aging was simulated by placing the samples in a dryer (Binder, Gleisdorf, Austria) with thermo-circulation of air. The samples were exposed to 70 °C for 14 days.

#### 2.2.10. Mechanical Properties

The mechanical strength tests of the vulcanizates were carried out with the usage of the Zwick Roell 1435 (Ulm, Germany) testing machine equipped with an extensometer. Five samples of dumbbell-shaped material were examined. The tests were performed according to the standard ISO 37 at a crosshead speed of 500 mm/min.

Based on changes in mechanical properties (before and after aging), the aging factor (K) was calculated according to Equation (1) [33]:

K = (TS · Eb)_after aging_ /(TS · Eb)_before aging_; (1)

TS—tensile strength [MPa]; Eb—the elongation at break [MPa]. Standard deviation for TS did not exceed +/−0.3 MPa and for EB +/−35%.

#### 2.2.11. Color Stability

The CM-3600d spectrophotometer (Konica Minolta, Sensing, Japan) was used to evaluate the color stability of vulcanizates during aging. The measurements were carried out in the spectral range of 360–740 nm. Before aging, all samples were examined and described by three components: L—brightness; a—color from green to magenta; b—color from blue to yellow. Then, after the aging processes, colorometric measurements were made for each sample. Based on the obtained changes, the total color changes of the natural rubber vulcanizates were determined according to Equation (2) [34]:

(2) $$∆E=\sqrt{{\left(∆L\right)}^{2}+{\left(∆a\right)}^{2}+{\left(∆b\right)}^{2}}$$

where ΔL—the change in brightness; Δa—the change in color from green to magenta; Δb—the change in color from blue to yellow. The measurements were performed five times for each aged and unaged sample. Standard deviation for ∆E did not exceed +/−0.2.

#### 2.2.12. The Cross-Linking Density of Vulcanizates

The equilibrium swelling (in toluene) method was used to calculate the cross-link density of vulcanizates. This parameter was determined based on the Flory–Rehner [35] (Equation (3)):

(3) $$\gamma_{e}=\frac{\ln\left(1-V_{r}\right)+V_{r}+{\mu V}_{r}^{2}}{V_{0}\left(V_{r}^{\frac{1}{3}}-\frac{V_{r}}{2}\right)}$$

$\gamma_{e}$—the cross-linking density (mol/cm^3^); V_0_—the molar volume of solvent (toluene: 106.7 cm^3^/mol); µ—the Huggins parameter of the polymer–solvent interaction, was calculated from Equation (4) [36]:

(4) $$\mu =\mu_{0}+\beta \cdot V_{r}$$

µ_0_—the parameter determining the number of non-crosslinked polymer/solvent relations; β—the parameter determining the number of cross-linked polymer/solvent relations (µ_0_ = 0.478, β = 0.228);V_r_—the volume fraction of the elastomer in the swollen gel (Equation (5)) [37].

(5) $$V_{r}=\frac{1}{1+Q_{w}\frac{\rho_{r}}{\rho_{s}}}$$

*ρ*_r_—natural rubber density (0.99 g/cm^3^); *ρ*_s_—solvent density (toluene-0.86 g/cm^3^); Q_w_—the weight of equilibrium swelling:

(6) $$Q_{w}=\left(\frac{m_{\mathrm{sw}}-m_{s}}{m_{s}}\right)\cdot \left(\frac{100+x}{100}\right)$$

m_sw_—the weight of the swollen sample; m_s_—the weight of the dry sample; 100—the elastomer content in the sample; x—the extract content in the sample.

In order to determine the Q_w_ parameter, samples (4) with a mass in the range of 30–50 mg were cut from the vulcanizate and soaked in toluene for 48 h (until the swelling equilibrium state was reached). After this time, the samples were weighed (m_sw_—the weight of the swollen sample). The swollen samples were dried to a constant weight and weighed (m_s_—the weight of the sample after drying).

## 3. Results and Discussion

### 3.1. Thermogravimetric Analysis (TGA)

**Figure 1:**
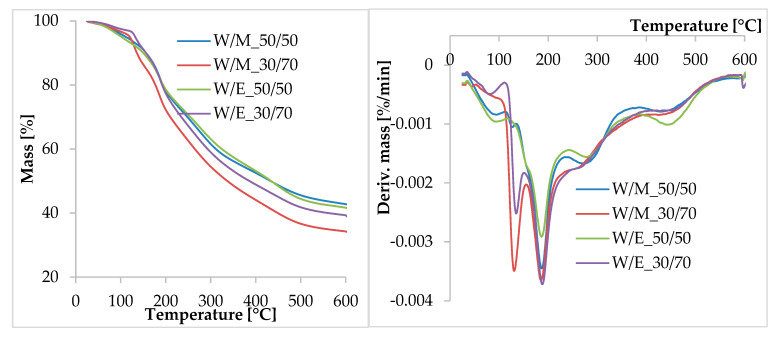
The TG and DTG curves of the natural extracts from *Equisetum arvense*.

Thermal decomposition of the freeze-dried extracts proceeded in several stages (Figure 1). The course of the thermal decomposition process, especially in the first steps, was different depending on the amounts of solvents used for the reaction. By analyzing the TG (thermogravimetry) and DTG (derivative thermogravimetry) curves in the temperature ranges of 25–120 °C and 120–140 °C, we saw a loss of weight related to the removal of residual solvents used for extraction. In the first range, the intensity of decomposition and the weight loss were greater for the extracts prepared in a mixture of water and alcohol with a 50/50 volume ratio. In the second range, the extracts prepared in a larger amount of alcohol were characterized by faster thermal decomposition and greater weight loss. The weight loss related to the content of bound solvent in samples of water/alcohol extracts (50/50 volume/volume) was about 8% (Table 2). In water/alcohol extracts (30/70 volume/volume) for methanol, it was approximately 11%, and for ethanol 7%. At higher temperatures, the thermal decomposition of extracts also proceeded in several stages, which waws confirmed by the formation of several peaks on the DTG curve in the temperature ranges: 140–350 and 350–600 °C. Thermal decomposition of many different secondary metabolites and active phenolic compounds contained in natural extracts could occur in this temperature range [38]. This is due to the fact that phenolic compounds are thermally stable up to the temperature of 200–250 °C [39], while flavonoids have a slightly lower thermal stability. In this range, chlorophyll can also undergo thermal degradation [40]. Hence, the highest intensity of thermal decomposition in this range occurred at 190 °C. The weight loss in this temperature range was the highest and ranged from 33% to 40%. Finally, from 350 to 600 °C, the sample was thoroughly degraded, leaving 35–40% residual weight, due to the non-oxidizing (argon) atmosphere used for the thermal analysis.

### 3.2. UV–Vis Diffuse Reflectance Spectroscopy

**Figure 2:**
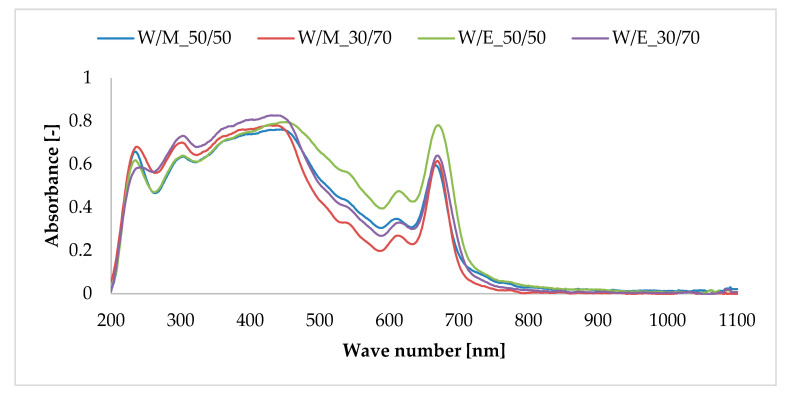
The UV–Vis spectrum of horsetail extracts.

Figure 2 presents the UV–Vis absorption spectra of horsetail extracts in the UV and visible light spectrum (200–1100 nm). In the visible range, the extracts’ spectra show peaks that are attributed to the presence of the chlorophyll pigment in the field horsetail. By analyzing the obtained spectrum, the absorption bands showing the maxima at 670 and 430 nm are related to the presence of chlorophyll “a.” Bands registered at 460 and 630 nm are characteristic for chlorophyll “b” [41]. The presence of an absorption band in the range of 400–450 nm indicated the carotenoid content in the obtained extract [42]. The carotenoids characteristic of horsetail species are β-carotene, β-cryptoxanthin, lutein epoxide and zeaxanthin [43]. According to the research on phenolic compounds, natural phenolic acids such as vanillic acid, gallic acid, caffeic acid and ferulic acid show strong absorption bands in the range of 270–320 nm [6,44]. Moreover, the absorption bands at 240–280 nm and 300–380 nm are characteristic of other active compounds present in field horsetail, such as flavonoids (kaempferol, quercetin, rutin and others) and related compounds [45,46,47]. Therefore, absorption peaks in the range of 350–220 nm found in the spectra of hydroalcoholic extracts confirm the presence of a mixture of active phenolic acids, flavonoids and related compounds contained in the samples.

### 3.3. Fourier Transform Infrared Spectroscopy (FTIR)

**Figure 3:**
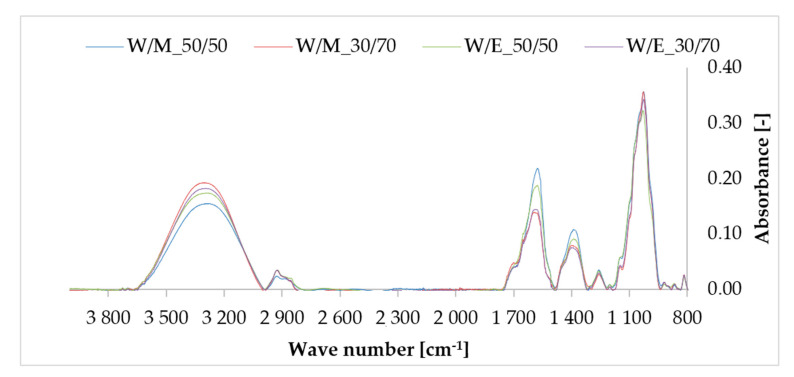
The FTIR spectrum of horsetail extracts.

By analyzing the FTIR spectra (Figure 3) of freeze-dried extracts of horsetail, several characteristic absorption bands can be distinguished. In the range of 3600–3000 cm^−1^, the absorption band was observed, corresponded to stretching vibrations of OH groups. The presence of this group could be due to the presence of water, alcohols, phenols and carbohydrates in the sample. Then, in the 3000–2800 cm^−1^ range, the band connected with asymmetric and symmetric stretching modes of C–H arising from methyl and methylene groups was recorded. The spectra also showed peaks in the regions 1750–1670 cm^-1^ (carbonyl) and 1680–1550 cm^−1^ (C=C bonds in the aromatic rings). The peak at 1390 cm^−1^ indicated O–H bending vibrations in the phenol group. According to Heneczkowski and colleagues [48] the ranges 1612–1598, 1570–1560 and 1488–1452 cm^−1^ are typical for the vibrations of the aromatic ring, characteristic of polyphenols contained in extracts. The sharp peaks in the region 1320–1000 cm^−1^ related to carbon–oxygen bonds (C–O) in the ether, esters and carboxylic acids are indicative of a wide variety of metabolites, such as tannins, flavonoids and anthraquinones, among others [49,50].

### 3.4. Total Phenolic Content (TPC) and Antioxidant Activity of Horsetail Extracts

There are many factors that influence the composition and antioxidant activity of a plant and its extracts, such as plant variety/cultivar; the part of the plant; the growing season; the particle size of the plant material; and the extraction method, including the type of solvent, extraction time and temperature [51]. In this study, different solvent types were tested for the extraction of bioactive compounds from *Equisetum arvense* L. herb. In our experiment, the extracts were prepared using water–methanol (30/70 volume/volume), water–ethanol (30/70 volume/volume), water–ethanol (50/50 volume/volume) and water–methanol (50/50 volume/volume). Polyphenols are a broad and diverse group of compounds that differ in chemical structure and polarity. It was documented in the literature that aqueous solutions of organic solvents give the highest extraction efficiencies of polyphenols [52,53]. Our studies showed statistically significant differences in TPC values (Table 3). The highest TPC was observed when W/M_ 50/50 was used (2355.94 µg_GAE_/mL) and the lowest when W/E_50/50 was used (1923.12 µg_GAE_/mL). No statistically significant differences in antioxidant activity measured with ABTS method were found. In DPPH assay, the highest antioxidant capacity showed the extract obtained with W/E_50/50 and the lowest when W/E_30/70 was used. No correlation between TPC and antioxidant activity (both in ABTS and DPPH methods) was observed. Generally, all extraction solvents gave high extraction efficiency with high TPC and high antioxidant capacity, and the differences were rather low.

### 3.5. Atomic Absorption Spectrometry (AAS)

Table 4 presents the results of metal (II) concentrations of horsetail extracts. The concentrations of Cd(II) and Pb(II) were very low. These metals (II) were probably in dry solid residues. Unfortunately in these residues were also natural rubber with high concentrations of cadmium(II) and lead(II). In the case of cobalt(II) the concentration was between 0.51 and 0.73 mg/kg and for nickel(II) it was in the range 3.52–5.20 mg/kg. Presence of Co(II) and Ni(II) in such amounts showed the formation of coordination compounds with organic ligands such as phenolic acids and flavonoids present in the horsetail. These organic ligands have a very good ability to coordinate with metal ions (II) due to their structures.

In Table 5 are the results of the determination of metal (II) concentrations in the elastomer vulcanizates filled with dry solid residues after the lyophilization process. All cases with additions of horsetail concentrations of metals (II) were very similar and lower than for the reference sample. In the unfilled system, the concentration of Pb(II) was 55.77 mg/kg; vulcanizates containing extracts were in the range 48.35–49.24 mg/kg. There was a similar situation was for cadmium(II) and nickel(II). In the control natural rubber sample, the concentrations were 5.53 and 0.63 mg/kg, respectively. In the filled vulcanizates, they were in the ranges 4.78–4.89 for Cd(II) and 0.55–0.58 mg/kg for Ni(II). The decreasing concentration values for these metals resulted from mixing natural rubber with horsetail. The lack of cobalt(II) in dry solid residues of horsetail extracts indicated that the coordination processes took place. These coordination compounds were contained in the extracts of horsetail, resulting in a better antioxidant activity of the measured extracts.

### 3.6. Influences of Aging Processes on the Properties of Vulcanizates

The existence of high level unsaturated isolated double bonds makes NR easily attacked by oxygen [54], especially under conditions of heat, pressure, light, etc. [55]. This would lead to the changes in mechanical and structural properties and external appearance (color, cracks and dullness). Thus, the thermo-oxidative performance is of key importance for the industrial applications of NR. Therefore, ensuring appropriate material strength, in the context of anti-aging resistance, is the basic goal that should be achieved when designing multifunctional elastomeric materials.

The main aging processes that affect the properties of natural rubber are oxidation, thermo-oxidation and photo degradation. In the first stage of oxidative and thermal-oxidative degradation, free radicals are produced on the NR chain as a result of hydrogen reception. It is a multi-stage process: First stage—reaction of the free radical with the oxygen molecule (O_2_) and formation of the peroxy radical (NR–O–O·). Second stage—separation of the hydrogen atom from another polymer chain and formation of a hydroperoxide (NR–O–O–H). Third stage—breakdown of the hydroperoxide into two new free radicals (NR–O· and ·OH) which separate different hydrogen and other polymer chains. Fourth stage—recombination or disproportion. In the case of photodegradation, the reactions are similar to those for thermal-oxidative degradation, provided that free radicals are directly generated by UV radiation. Then, chain breaks, cross-linking and secondary oxidation may occur [56].

Field horsetail extracts, showing high antioxidant potential, have been used as natural anti-aging additives. Due to the fact that elastomeric materials are exposed to various environmental factors during their use, the influences of three types of accelerated simulated aging processes on the properties of natural rubber containing plant extracts were investigated. In order to assess the resistance of natural vulcanizates to UV and SOLAR radiation at increased humidity and temperature, and thermo-oxidative conditions, the following tests were carried out on changes (before and after aging processes):

- Spatial structure (cross-linking density);
- Functional properties (mechanical properties);
- Physical properties (color).

#### 3.6.1. The Aging Effects on the Changes in Cross-Linking density

**Figure 4:**
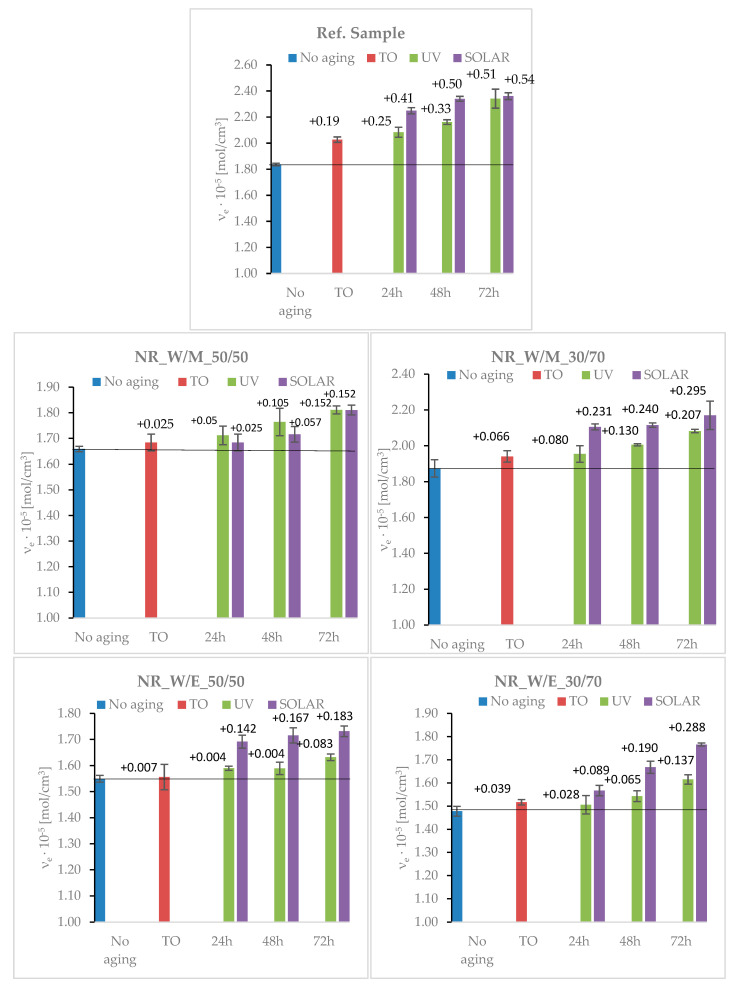
The cross-linking densities of vulcanizates before and after simulated aging processes: thermo-oxidative (TO), UV and SOLAR.

Under the influences of UV radiation, increased temperature and the presence of oxygen, natural rubber is exposed to reactions leading to the degradation of its structure. This mainly lead to radical formations and to the chain reactions up to either cross-links or chain-scissions [56]. When analyzing the data presented in Figure 4 concerning changes in cross-linked density before and after the aging processes, a significant increase in the value of ν_e_ was observed for aged reference samples. Cross-linking density increased with aging, clearly indicating the formation of an extra cross-linking network. The solar-aged sample had the highest growth rate and the highest final cross-linking density. The increase of the νe value for vulcanizates subjected to solar aging ranged from 0.41 × 10^−5^ mol/cm^3^ for SOLAR_24 to 0.54 × 10^−5^ mol/cm^3^ for SOLAR_72. In the case of the reference sample, the cross-linking density after thermo-oxidative aging (TO) increased by 0.19 × 10^−5^ mol/cm^3^. Slightly greater changes were observed after UV aging.

The increase in cross-linking density after aging results from the degradation of natural rubber through undesirable and uncontrolled reactions of destroying the polymer chains. As a consequence of the changes, chain scission and the formation of chain molecules occur, which leads to a reduction in the molecular weight of the elastomer. Additionally, such polymer chains are easily entangled, limiting the mobility of macromolecules and reducing their flexibility. Moreover, this leads to a reduction in swelling and hence an increase in the cross-linking density and stiffness of the vulcanizates. Furthermore, secondary cross-linking reactions may occur due to the elevated temperature. Part of the unreacted sulfur cross-linker at elevated temperature can actively participate in further cross-linking reactions, leading to the formation of new network nodes.

The effect of the addition of natural horsetail extracts on the protection of natural rubber against aging is illustrated in Figure 4. The presented data clearly shows that the increase in cross-linking density after aging for all vulcanizates with the addition of extracts was significantly lower than in the case of the reference sample. However, there were no significant differences in the effect of the type (amount and type of solvents used) of extracts on changes in cross-linking density. Nevertheless, extracts prepared with the volume fraction of water/alcohol_30/70 solvents were characterized by the lowest anti-aging activity in terms of cross-linking density. In the case of vulcanizates containing these extracts, increases in the concentration of network nodes by approximately 0.07 × 10^−5^ mol/cm^3^ (NR_W/M_30/70) and 0.04 × 10^−5^ mol/cm^3^ (NR_W/E_30/70) were recorded after thermo-oxidative aging compared to the unaged sample.

Analyzing other types of aging, also for these materials, the maximum increase in cross-linking density after SOLAR_72h aging was 0.29 × 10^−5^ mol/cm^3^ (NR_W / M_30 / 70), while after UV_72 it was 0.21 × 10^−5^ mol/cm^3^. As can be seen from the attached data, changes in the spatial structure of vulcanizates containing natural antioxidants in the form of horsetail extracts were much lower. Plant phenols are multifunctional and can act as reducing agents (free radical terminators), metal chelators and singlet oxygen quenchers [57]. Thanks to this, the compounds can stabilize the activity of natural rubber degradation processes leading to structural changes in the spatial network of vulcanizates.

#### 3.6.2. The Aging Effect on the Changes in Mechanical Properties

A key aspect of materials in the context of long-term use is the influence of progressive degradation on the performance and functional properties of materials. For this purpose, the mechanical properties of the reference test and elastomers with the addition of natural extracts for tensile strength (TS) and elongation at break (Eb) before and after the aging processes were examined. The aging factor “K” was determined from the changes in these values.

**Figure 5:**
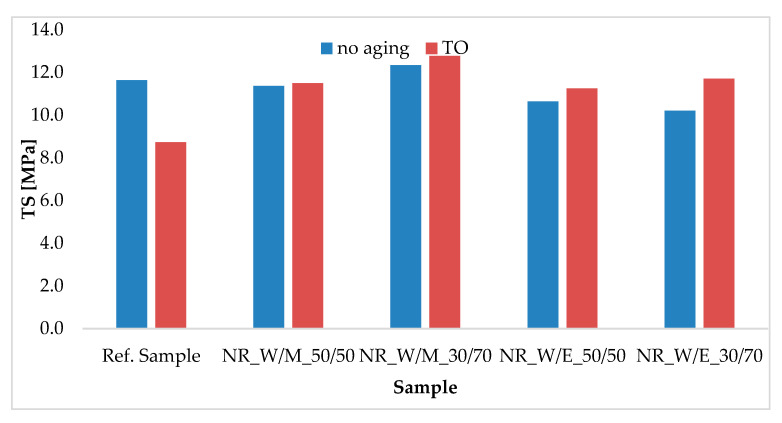
Tensile strength of vulcanizates before and after simulated thermo-oxidative aging process.

Figure 5 and Table 6 show the TS and Eb values obtained for the tested elastomeric vulcanizates before and after simulated thermo-oxidative aging, and the K coefficient. The unaged sample of pure natural rubber reached the value of 11.6 MPa. Application of horsetail extracts slightly affected the mechanical strength values; TS natural vulcanizates filled with the extract ranged from 10 to 12 MPa. The accelerated thermoxidative aging simulation process made the biggest changes to the reference sample. The tensile strength was reduced to 8.7 MPa. In the case of elastomer containing natural antioxidants, the value did not change or increased slightly. When describing the effect of cross-linking density on mechanical properties, it can be stated that the tensile strength increases with increasing network node concentration, until the critical value of cross-linking density is reached. In the case of the reference test, the increase in density was so large that it significantly stiffened the vulcanizate, leading to a reduction in elongation at break (Table 6), and consequently a decrease in mechanical strength. For natural rubber vulcanizates with the addition of extracts (regardless of the type), a significantly smaller decrease in the Eb value after aging TO was observed compared to the unfilled sample. As a result of changes in mechanical properties, the aging coefficient for the reference sample was 0.69. On the other hand, for natural rubber containing anti-aging bio-additives, it was close to unity, which proved very small changes in mechanical properties and active protection against thermo-oxidative factors.

**Figure 6:**
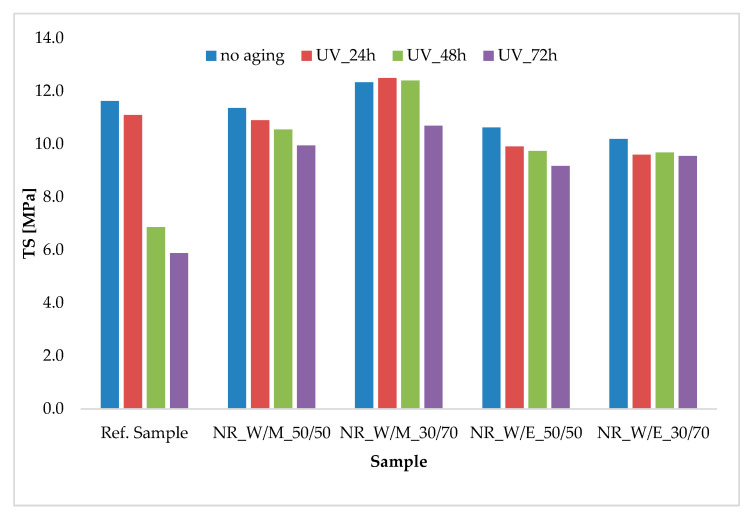
Tensile strength of vulcanizates before and after simulated UV aging process.

Changes in mechanical properties after simulation of accelerated UV aging on the tested samples are shown in Figure 6 and Table 7. For all vulcanizates, the TS and Eb values decreased with the progressive time of exposure of the sample to UV radiation. As in the case of the TO aging, the greatest differences in the strength values after UV aging were observed for the reference sample. The TS value decreased successively to 11.1, 6.9 and 5.9 MPa after 24, 48 and 72 h UV aging, respectively (Figure 6). Large drops in mechanical strength are the result of changes in the spatial structure of vulcanizates (cross-linking density) leading to premature failure of the sample and reduction of elongation at break (Table 7). Accordingly, the unfilled natural rubber after UV aging was characterized by low values of the K coefficient. The K value for the control sample after 72 h of aging was only 0.4. In the case of vulcanizates containing anti-aging active extracts, changes in the energy of mechanical deformation were small. The K coefficients obtained at the level of 0.9–0.8 even after 72 h exposure were the proof. It is worth mentioning that horsetail extracts were characterized by a high absorption of UV radiation and thus act actively as a means of limiting the aging of elastomer materials.

**Figure 7:**
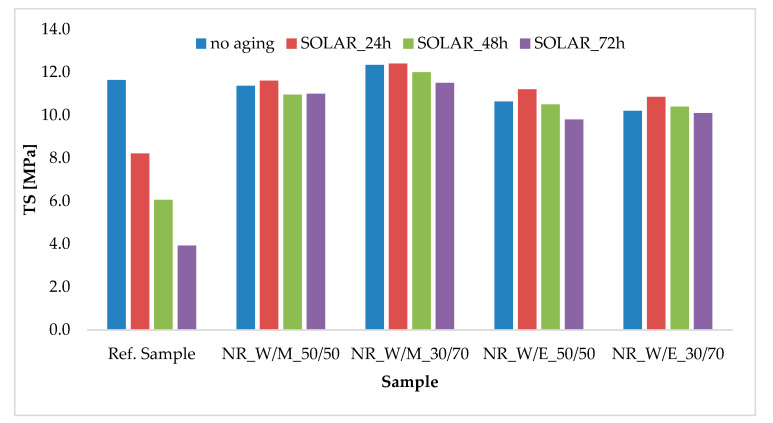
Tensile strengths of vulcanizates before and after the simulated SOLAR aging process.

Increased temperature and exposure to solar radiation in the full spectrum are the most typical conditions to which polymer materials are exposed during their use. Among the examined factors, they caused the greatest changes in mechanical properties in natural rubber vulcanizates (Figure 7 and Table 8). As with the previous types of aging, solar aging significantly reduced TS and Eb values for the reference sample, resulting in low K-values (0.62, 0.38 and 0.24 for SOLAR_24h, SOLAR_48h and SOLAR_72H, respectively). On the other hand, vulcanizates containing natural extracts (regardless of the type) showed high anti-aging resistance. The value of the K coefficient for these materials did not drop below 0.87. Comparing the samples before and after solar aging, the decrease in tensile strength did not exceed 1 MPa (Figure 7), and in the case of the Eb value, the changes were several dozen percent.

#### 3.6.3. The Aging Effect on the Changes in Color

As confirmed by UV–Vis spectroscopy studies, horsetail extracts contained natural pigments such as chlorophyll and carotenoids. The presence of these compounds (mainly chlorophyll) made the natural rubber vulcanizates colorful. Color stability is one of the physical properties that determine the useful life of elastomeric materials in many applications. In order to determine the resistance of the tested materials after aging processes, the color changes in vulcanizates were determined based on the CIE-Lab scale. The results of these studies are summarized in Table 9. Thermo-oxidative aging caused the lowest color changes in all tested materials. Among the vulcanizates, the reference sample showed the highest value of ΔE, and the largest differences in color before and after TO aging. Natural rubber materials containing extracts prepared in water/methanol showed slightly less color stability than those based on water / ethanol. In most cases, UV and SOLAR aging resulted in greater changes in the ΔE parameter compared to TO degradation. For all vulcanizates, increasing the exposure time of the samples to the agent, causing accelerated degradation, adversely affected the color stability. In both types of aging (UV and SOLAR), the reference sample showed the lowest anti-aging resistance in terms of color change. The samples with the addition of natural antioxidants showed increased resistance of the material to color changes, especially in the case of solar aging. Moreover, the vulcanizates with the addition of extracts—water/alcohol_30/70 showed slightly better color stability after UV and SOLAR aging.

The antioxidant effect of horsetail extracts results from the presence of active compounds such as polyphenols, phenolic acids and flavonoids. Phenolic compounds, acting as antioxidants, react with various free radicals. The mechanisms of their antioxidant activity include hydrogen atom transfer, single electron transfer, sequential electron transfer with proton loss, and transition metal chelation [58]. Summarizing the activity of extracts to anti-aging properties in natural rubber, a clear improvement in the resistance of vulcanizates to external aging accelerating factors was obtained. The effect of specific extract components is reflected in the stability of the material, which was determined on the basis of changes in color, cross-linking density and strength parameters.

## 4. Conclusions

The thermal decomposition of freeze-dried horsetail extracts indicated the presence of various secondary metabolites, especially phenolic acids and flavonoids. The analysis of the UV–Vis absorption spectra of bioadditives in the UV and Visible light spectrum (200–1100 nm), and the FTIR spectra confirmed the presence of a mixture of active phenolic acids, flavonoids and related compounds in the samples. The analysis of selected heavy metals in the tested materials via Atomic Absorption Spectrometry (AAS) showed their presence. Phenolic acids and flavonoids have the ability to chelate metals, which may also support the assumptions about the anti-aging properties of biomaterials. Undoubtedly, the studies of Total phenolic content (TPC) and antioxidant activity of *Equisteum arvense* freez-dryed extracts determined by the DPPA and TBPS methods were undeniable evidence of the high antioxidant potential of the tested compounds.

The evaluation of the resistance of natural vulcanizates to thermo-oxidative, ultraviolet and solar aging processes was carried out on the basis of examining changes (before and after aging processes) in cross-linking density, mechanical properties and color. The study of the spatial structure of vulcanizates containing natural antioxidants in the form of horsetail extracts showed high resistance of these materials to simulated aging processes. The increase in cross-linking density of these samples was small by about a few percent, compared to the reference sample, where the υe value increased even by about 30% in relation to the unaged material. The highest resistance in terms of cross-linking density was shown by vulcanizates subjected to the process of thermo-oxidative degradation, then ultraviolet degradation, and the lowest by solar degradation, although the results were still satisfactory. As in the case of the cross-linking density analysis, the materials with the addition of *Equisteum arvense* extracts showed increased aging resistance in terms of mechanical strength. The analysis of the color change of natural rubber vulcanizates confirmed the previous conclusions. The unfilled samples showed a tendency to lose color as the aging processes progressed. The materials with the addition of freeze-dried extracts were characterized by a much higher resistance to color changes of the tested samples. Thermo-oxidative aging had the least influence on the color changes, slightly higher UV and solar aging.

## Figures and Tables

**Table 1:** Formulations of rubber mixtures.

Component	Ref. Sample	NR_ W/M_50/50	NR_ W/M_30/70	NR_ W/E_50/50	NR_ W/E_30/70
**NR (phr)**	100				
**W/M_50/50 (phr)**	-	5	-	-	-
**W/M_30/70 (phr)**	-	-	5	-	-
**W/E_50/50 (phr)**	-	-	-	5	-
**W/E_30/70 (phr)**	-	-	-	-	5
**Zinc oxide (phr)**	5				
**2-mercaptobenzothiazole (phr)**	2				
**Stearic acid (phr)**	1				
**Sulphur (phr)**	2				

**Table 2:** Percentage mass losses of horsetail extracts (Δm) in various temperature regimes and the residue after thermal decomposition (R_600_).

Sample	Δm_25–140 °C_ [%]	Δm_140–350 °C_ [%]	Δm_350–600 °C_ [%]	R_600_ [%]
W/M_50/50	7.7	36.0	13.5	42.8
W/M_30/70	11.3	40.1	14.4	34.2
W/E_50/50	8.7	33.8	15.8	41.6
W/E_30/70	7.0	39.9	13.9	39.3

**Table 3:** Characteristics of natural horsetail extracts in terms of the total phenolic content and antioxidant activity. The results are expressed as mean ± SD.

Solvent Type	TPC [µg_GAE_/mL]	Antioxidant Activity [µg_TE_/mL]	
		ABTS	DPPH
W/M_50/50	2355.94 ± 81.21 ^a^	606.18 ± 0.53 ^a^	932.71 ± 3.90 ^a.b^
W/M_30/70	2211.38 ± 9.05 ^b^	615.67 ± 1.40 ^a^	937.31 ± 4.16 ^a.b^
W/E_50/50	1923.12 ± 44.44 ^c^	609.80 ± 16.35 ^a^	940.25 ± 4.16 ^a^
W/E_30/70	2120.81 ± 56.30 ^b^	618.70 ± 9.49 ^a^	924.44 ± 1.56 ^b^

^a,b,c^—statistically significant differences (*p* < 0.05).

**Table 4:** Concentrations of metals(II) in extract samples.

Sample	Co [mg/kg]	Ni [mg/kg]	Cd [mg/kg]	Pb [mg/kg]
W/M_50/50	0.71	5.20	0.03	0.06
W/M_30/70	0.65	3.52	0.03	0.07
W/E_50/50	0.73	4.74	0.03	0.07
W/E_30/70	0.51	3.79	---	0.08

**Table 5:** Concentrations of metals (II) in samples of dry solid residues after lyophilization process.

Sample	Co [mg/kg]	Ni [mg/kg]	Cd [mg/kg]	Pb [mg/kg]
Ref. sample	-	0.63	5.53	55.77
NR_W/M_50/50	-	0.58	4.79	47.43
NR_W/M_30/70	-	0.56	4.89	48.85
NR_W/E_50/50	-	0.57	4.83	48.35
NR_W/E_30/70	-	0.55	4.78	49.24

**Table 6:** Elongation at break of vulcanizates before and after simulated thermo-oxidative aging process and aging factor (K).

Sample	Before Aging	TO	
		After Aging	K[-]
	Eb [%]	Eb [%]	
**Ref. Sample**	734	670	0.69
**NR_W/M_50/50**	797	758	0.96
**NR_W/M_30/70**	781	699	0.93
**NR_W/E_50/50**	748	678	0.96
**NR_W/E_30/70**	773	721	1.07

**Table 7:** Elongation at break of vulcanizates before and after simulated UV aging process and aging factor (K).

Sample	Before Aging	UV_24h		UV_48h		UV_72h	
		After Aging	K[-]	After Aging	K[-]	After Aging	K[-]
	Eb [%]	Eb [%]		Eb [%]		Eb [%]	
**Ref. Sample**	**734**	**721**	0.94	679	0.55	623	0.43
**NR_W/M_50/50**	797	790	0.95	783	0.91	774	0.85
**NR_W/M_30/70**	781	780	1.01	769	0.99	739	0.82
**NR_W/E_50/50**	748	760	0.95	775	0.95	777	0.90
**NR_W/E_30/70**	773	778	0.95	782	0.96	756	0.92

**Table 8:** Elongation at break of vulcanizates before and after simulated SOLAR aging process and aging factor (K).

Sample	Before Aging	SOLAR_24h		SOLAR_48h		SOLAR_72h	
		After Aging	K[-]	After Aging	K[-]	After Aging	K[-]
	Eb [%]	Eb [%]		Eb [%]		Eb [%]	
**Ref. Sample**	**734**	**644**	0.62	543	0.38	524	0.24
**NR_W/M_50/50**	797	778	1.00	789	0.95	759	0.92
**NR_W/M_30/70**	781	725	0.93	732	0.91	731	0.87
**NR_W/E_50/50**	748	732	1.03	736	0.97	730	0.90
**NR_W/E_30/70**	773	775	1.07	744	0.98	704	0.90

**Table 9:** The values of color change (ΔE) of the natura rubber vulcanizates filled with *Equisteum arvense* extracts exposed to various aging factors.

ΔE [-]							
Sample	TO	UV			SOLAR		
		24 h	48 h	72 h	24 h	48 h	72 h
**Ref. Sample**	5.51	5.02	8.58	9.26	7.13	8.92	10.85
**NR_W/M_50/50**	3.20	3.60	5.51	5.60	5.51	5.51	6.50
**NR_W/M_30/70**	3.11	4.23	6.57	7.28	2.35	3.72	3.74
**NR_W/E_50/50**	2.92	2.85	3.67	4.77	2.42	4.09	4.21
**NR_W/E_30/70**	2.79	3.85	4.29	5.24	1.21	2.46	2.75
